# “Moving beyond silos”: focus groups to understand the impact of an adapted project ECHO model for a multidisciplinary statewide forum of substance use disorder care leaders

**DOI:** 10.1186/s13722-024-00485-3

**Published:** 2024-08-08

**Authors:** NithyaPriya Ramalingam, Eowyn Rieke, Maggie McLain McDonnell, Emily Myers, Dan Hoover

**Affiliations:** 1 Department of Medicine, Section of Addiction Medicine, Oregon Health & Science University, Portland, OR USA; 2 Fora Health, Addiction Treatment Center, Portland, OR USA; 3 Oregon ECHO Network (OEN), Oregon Health & Science University, Portland, OR USA; 4 Oregon Rural Practice-based Research Network (ORPRN), Oregon Health & Science University, 3181 S.W. Sam Jackson Park Road, Mail Code: UHN 30, Portland, OR USA; 5 Project ECHO (Extension for Community Healthcare Outcomes), Oregon Health & Science University, Portland, OR USA; 6 Recovery Works Northwest, Portland, United States

**Keywords:** ECHO model, Substance-related disorders, Case-based learning, Continuing medical education, Substance use treatment

## Abstract

**Background:**

Although clinical substance use disorder (SUD) care is multidisciplinary there are few opportunities to collaborate for quality improvement or systems change. In Oregon, the Project ECHO (Extension for Community Healthcare Outcomes) model was adapted to create a novel multidisciplinary SUD Leadership ECHO. The objective of this study was to understand the unique effects of the adapted ECHO model, determine if the SUD Leadership ECHO could promote systems change, and identify elements that enabled participant-leaders to make changes.

**Methods:**

Four focus groups were conducted between August and September of 2022 with a purposive sample of participants from the second cohort of the Oregon ECHO Network’s SUD Leadership ECHO that ran January to June 2022. Focus group domains addressed the benefits of the adapted ECHO model, whether and why participants were able to make systems change following participation in the ECHO, and recommendations for improvement. Thematic analysis developed emergent themes.

**Results:**

16 of the 53 ECHO participants participated in the focus groups. We found that the SUD Leadership ECHO built a multi-disciplinary community of practice among leaders and reduced isolation and burnout. Three participants reported making organizational changes following participation in the ECHO. Those who successfully made changes heard best practices and how other organizations approached problems. Barriers to initiating practice and policy changes included lack of formal leadership authority, time constraints, and higher-level systemic issues. Participants desired for future iterations of the ECHO more focused presentations on a singular topic, and asked for a greater focus on solutions, advocacy, and next steps.

**Conclusions:**

The adapted ECHO model was well received by focus group participants, with mixed reports on whether participation equipped them to initiate organizational or policy changes. Our findings suggest that the SUD Leadership ECHO model, with fine-tuning, is a promising avenue to support SUD leaders in promoting systems change and reducing isolation among SUD leaders.

## Background

COVID-19 emerged in the midst of a relentless overdose crisis. The syndemics of COVID-19 and the overdose crisis created an urgent, unique need for increased communication and collaboration within SUD treatment [1]. Diverse disciplines participate in clinical SUD care, but opportunities to collaboratively advance quality improvement or systems change are rare [2]. In addition, quality improvement in health care settings is typically slow and methodical. Change often relies on published evidence, which lags behind real-time needs [3, 4]. COVID-19 and the arrival of non-pharmaceutical fentanyl on the West Coast prompted a need for urgent expansion and adaptations in SUD care delivery [5, 6] such as telehealth services for low-barrier access to medications [7]. Nationally, there has been a windfall of new addiction medicine legislation in the wake of COVID-19 and the continued opioid crisis [8]. In Oregon, the landmark Drug Addiction Treatment and Recovery Act was passed to decriminalize small-quantity drug possession and expand funding for SUD services [9].These rapid changes have two implications. First, Oregon’s SUD providers were caught in a rapidly changing landscape, and it was unclear how local and organization level systems would adapt to higher-level shifts. Second, SUD providers nationally may have increased opportunities to be involved directing reform in a time when the public eye is turning to overdose and SUD [10].

As SUD care providers adapted their care delivery to meet the ongoing needs of these co-existing crises, addiction medicine faculty at Oregon Health and Science University (OHSU) re-purposed the Project Extension for Community Healthcare Outcomes (ECHO) model to support SUD care leaders in this time of turmoil [11]. ECHO traditionally uses case-based tele-mentoring to equip health professionals to deliver best-practice care and has been implemented to address multiple medical conditions, including for substance use disorder. ECHOs recruit broadly via email listservs, meet virtually in one-hour weekly sessions, and combine didactic curriculum with case presentations. Curriculum topics are set months in advance of the program based on the expert faculty team’s choice of priority topics and previous participant feedback. De-identified patient or systems-level cases are presented by participants and case discussions conclude with recommendations by the ECHO faculty experts and participants in the session. The SUD Leadership ECHOs were distinctly different than traditional ECHO programs; unique goals required a special development process, individual recruitment of participants, curated session content, and a modified once-monthly session format. To address the aforementioned need for increased collaboration, the SUD Leadership ECHO was designed to include participants from different roles and leadership levels within SUD treatment and harm reduction. The ECHO’s development team hypothesized that the differing perspectives and multi-level leadership nature of the group would help to promote novel learning and systems change.

In partnership with the Oregon ECHO Network (OEN), a statewide utility that supports ECHO programming in Oregon, OHSU Addiction Medicine faculty launched a 12-session *SUD COVID Response ECHO* to convene Oregon’s leaders in SUD care, identify barriers to effective care, and pioneer solutions together [12, 13]. The program launched in April 2020 and focused primarily on COVID-related changes within the SUD treatment system. Following the success of the SUD COVID Response ECHO, the SUD Leadership ECHOs cohort 1 and 2 launched in September 2020 and January 2022 respectively. These iterations featured broader learning objectives: to brief leaders about emerging SUD issues in Oregon, explore solutions, and motivate systems improvement.

Clinical outcomes from ECHO programs have previously been evaluated [14, 15], however, higher-level systems may remain slow or unaware of change directed by ECHO. Therefore, this study aimed to understand the flexibility of the ECHO model, determine if the SUD Leadership ECHOs could drive systems change, and identify what factors may enable participant-leaders to make changes.

## Methods

This qualitative study used focus groups to understand how participants benefited from their involvement in the SUD Leadership ECHO. The study was conducted by a multidisciplinary team with expertise in SUD clinical care, ECHO program management, and qualitative methods as a partnership between the Oregon Rural Practice-based Research Network, the OEN, and the OHSU Section of Addiction Medicine. Data collection and analysis occurred from August to November 2022 and focused on the SUD Leadership ECHO’s cohort 2. The study was deemed “not human subject research” by the OHSU Institutional Review Board.

### Intervention description

The SUD Leadership ECHO cohort 2 was comprised of six sessions. Cohort 2 topic and description are listed in Table 1 below. One-hour sessions were hosted on Zoom and were divided in halves for a didactic presentation and a systems case discussion. Recruitment for cohort participants was an intensive iterative process, by invitation only. The goal of recruitment was to enroll SUD care leaders across the state of Oregon diversely representing ethnicities, gender identities, geographic and organizational settings, and experiential knowledge: clinicians, pharmacists, payor representatives, harm reductionists, persons who use drugs, advocates, and public health professionals. Table 2 provides further descriptions of leadership types considered for recruitment.

**Table 1:** Session topics of SUD leadership ECHO cohort 2

	Didactic Topic	Didactic Presenter(s)	System Case Topic	System Case Discussion Summary
Session 1-January 2022	**Interviews with People Who Use Drugs in Oregon: Fentanyl Results.** Report of 34 structured interviews with people who use drugs and their use of fentanyl. Themes included changed behaviors around fentanyl use and fentanyl purchasing, overdose experiences, and overdose responses.	#1: Peer specialist and crisis manager #2: Research associate specializing in SUD	Increasing Oregon’s behavioral health workforce	A behavioral health manager from a Coordinated Care Organization (Oregon’s version of an Accountable Care Organization) shared challenges with recruiting and retaining its behavioral health workforce. The presenter shared strategies the organization is doing to address workforce concerns including telehealth continuation and expansion, prioritizing high need populations, and investing in new payment models. Recommendations included increasing the budget for workforce recruitment, reducing requirements for CADC credentialing, and incentivizing continuing education and additional virtual training opportunities.
Session 2-February 2022	**Staff Well-being**. Presenter shared her organization’s strategies to improve staff morale and connect with staff remotely due to COVID 19. Key topics included the role of planned staff connection time at virtual clinical meetings and the role of formal supervision to provide structured support meetings for the SUD workforce.	Chief clinical officer at a SUD treatment organization	Buprenorphine access barriers at Oregon pharmacies	An Oregon Health Authority leader shared about challenges related to buprenorphine access through community-based pharmacies. Access is jeopardized due to long wait times, pharmacy closures, and controlled substance quotas which can be self-imposed by pharmacies or perceived due to oversight by coordinated care organizations, wholesale suppliers, or the DEA. Participants were asked to comment on how buprenorphine shortages are affecting their communities and innovate solutions. These solutions included using mail order suppliers, clarifying and removing quota barriers (real or perceived), and health authority support of replacement services after pharmacy closures.
Session 3-March 2022	**SUD Care in Jail Setting: Example of Clackamas County Jail.** Presenter shared jail policies and county progress to address SUD and OUD, particularly the hiring of an MOUD care coordinator for the jail. Presenter identified barriers to further progress including lack of SUD treatment funding, recovery housing in the county, workforce shortages within jail healthcare, short jail stays, and regulations upon MOUD.	Healthcare administrative services manager at a large county jail	Methadone access after hospital discharge	A hospital-based addiction medicine physician shared about barriers to continuing methadone at hospital discharge, particularly for patients with limited mobility or discharging to a skilled nursing facility (SNF). Usual interpretation of regulations requires an in-person evaluation at the opioid treatment program (OTP) after discharge, even if the patient is stable having completed a methadone induction in the hospital under addiction medicine expert supervision. The discussion explored the regulations upon new intakes to opioid treatment programs and adaptations by SNF staff and OTP staff that could facilitate methadone in SNFs. An ECHO participant who is a local Coordinated Care Organization leader hosted a follow-up bonus session three months later to convene key stakeholders and further develop a pilot program for methadone access at a SNF.
Session 4-April 2022	**Integrating Somatic-based Care into SUD Treatment.** Presenter reviewed the connection between adverse childhood events (ACEs) and SUD, acknowledged that traditional treatment models may not be effective for those with SUD and trauma, suggested that somatic care (e.g. meditation, tai chi) is a model to consider.	Addiction medicine physician at a SUD treatment organization	Alcohol regulation and taxation	The director of an Oregon-based recovery advocacy organization presented on the impacts of alcohol consumption in Oregon, alcohol taxation, and the effects of alcohol industry lobbying. Three bills were introduced during the 2021 Oregon state legislative session to adjust regulations, consider raising taxation, and fund prevention and treatment. These bills were defeated by lobbying by the alcohol industry. ECHO participants were asked to rally public health leadership to oppose the alcohol lobby. In the discussion, healthcare providers shared concerns for the effects that raising alcohol taxation could have upon individuals with alcohol use disorder, including substitution with toxic non-beverage alcohols.
Session 5-May 2022	**Psilocybin and SUD.** Oregon’s ballot measure 109 established a psilocybin regulatory process to facilitate psilocybin treatment programs. Presenter shared the pharmacology of psilocybin, long term effects, potential positive effects on trauma, as well as an overview of the Oregon Health Authority’s Psilocybin Program in development.	Vice chair of the Oregon Psilocybin advisory board and program manager for Measure 110	Emerging adolescent non-pharmaceutical fentanyl use	An adolescent psychiatrist working in an inpatient SUD treatment setting shared about emerging non-pharmaceutical fentanyl use in adolescents and poor access to care. Recently multiple youth SUD treatment programs had closed. The ECHO participants were asked to brainstorm policy solutions to support more sustainable youth SUD services and create a referral network for youth to access buprenorphine. Solutions included referring to a low-barrier tele-buprenorphine clinic serving ages 16 and older, referring to family medicine clinics, and expanding admissions at community withdrawal management centers to include adolescents. One guest participant reported in follow up that they had launched a school-based buprenorphine clinic. The presenting adolescent psychiatrist was recruited to facilitate a new *SUD in Adolescents* ECHO program for Winter quarter 2023.
Session 6-June 2022	**Measure 110 Implementation Update.** Shared updates about implementation of Oregon ballot measure 110 (the Drug Addiction Treatment and Recovery Act) that decriminalized possession of small amounts of drugs, allocated additional funding for addiction recovery and harm reduction services, and organized funded partner organizations into behavioral health networks for each of Oregon’s counties.	#1: Tri-Chair of Measure 110 oversight and accountability council; outreach director at recovery advocacy nonprofit #2: Tri-Chair of Measure 110 oversight and accountability council; harm reduction peer specialist at county health department	Non-pharmaceutical fentanyl and community overdose response	A national harm reduction expert based in Portland presented changes in syringe service program utilization and overdose response due to the arrival of widespread non-pharmaceutical fentanyl. Participants were asked about policy changes that could respond to the introduction of fentanyl. Naloxone distribution, adjustments to buprenorphine inductions, increased access to pharmacotherapies for SUD, and novel models of overdose response were discussed.

**Table 2:** Definitions and examples of leadership categories

Leadership Level	Definition	Examples
Direct Care	Performs direct care of individuals in a professional context and does not lead a SUD care program. May be an informal leader among colleagues or a leader in community SUD associations outside of their primary employment context.	A behavioral health provider at a large health system. A pharmacist at a rural clinic who is a specialist in SUD pharmacotherapies.
Organizational or Program	Leads a SUD services program within their workplace or participates in leadership of an organization focused on SUD services. May also perform direct care of individuals.	A behavioral health program manager at a rural SUD services organization. A chief medical officer at a multi-site SUD and MOUD services clinic.
State or Regional systems	Employed by an organization that functions at the state or county level to direct the care of persons with SUD. May also perform direct care of individuals. Includes staff of accountable care organizations that manage Medicaid, termed *coordinated care organizations* in Oregon.	A manager at a non-profit advocacy group. A harm reduction project manager with County Public Health. Coordinated care organization personnel.

### Participants

Direct outreach to attendees of at least one session of cohort 2 (*n* = 53) was conducted via email by the principal investigator (DH).

### Data collection

A semi-structured, 15 question guide was developed by the qualitative analyst (NR) and explored SUD leadership ECHO participants’ experiences. The guide was iteratively refined by the larger study team as data collection progressed based on de-briefing of early focus groups. The focus group guide can be found in Appendix A.

Four focus groups were conducted by an experienced qualitative analyst (NR) by Zoom videoconference. Focus groups lasted an average of 51 min (range 40–58), contained an average of 4 participants (range 2–6), were digitally recorded with verbal consent, and were transcribed professionally. Transcriptions were validated, de-identified, and assigned a participant ID by a qualitative analyst (EM). Data was monitored for saturation (e.g., participants presented no new information), at which point recruitment stopped [16].

Transcripts were uploaded to Atlas.ti for data management and analysis. Data was analyzed concurrently using Braun and Clarke’s approach to thematic analysis [17]. An initial code book was developed using a combination of deductive and inductive codes. The code book was tested on a subset of transcripts and coded by a second analyst (EM) to ensure reliability; it was iteratively refined through analytic team meetings which included qualitative analysts (NR, EM), OEN director (MMM), and the ECHO’s project coordinator (KG). Transcripts were then dual-coded by qualitative analysts (NR, EM) using the finalized code book. Emergent themes were identified in a dialogue-based refinement process.

### Demographic information

Participant information such as age, credential, location, and type of practice was collected from ECHO registration data. The Addiction Medicine ECHO Program director (DH) and the OEN director (MMM) collaboratively classified participants’ organizations into leadership categories as described in Table 2.

## Results

16 individuals participated in the focus groups out of 53 SUD Leadership ECHO participants. Focus group participants were representative of the overall ECHO participants, as their demographics were similar as seen in Table 3 below. Focus group participants represented 7 of the 36 counties in Oregon, with 25% of participants practicing in rural areas. On average, focus group participants attended 4.5 of the sessions as compared to 3.4 sessions attended by overall participants. Focus group participants were mainly classified as Organization/Program leaders (50%) or State/Regional system leaders (38%), and had diverse credentials. Detailed descriptions and example roles for each category of leadership are included in Table 2. They represented a wide variety of settings, including large health systems, specialty addiction and behavioral health treatment, primary care, accountable care organizations, the state health authority, and county health departments.

**Table 3:** Characteristics of cohort 2 participants (*n* = 53) and focus group participants (*n* = 16)

	ECHO Cohort		Focus Groups	
	Number	%	Number	%
**Cohort 2 Enrollment**				
Registered online	68			
Approved registrants	61	90%	16	
Participants (attended ≥ 1 session)	53	87%	16	100%
Repeat participants from Cohort 1	20	38%	7	44%
First time participants in an Oregon ECHO Network program	27	51%	4	25%
Average number of sessions attended	3		4	
**Gender of Participants**				
Woman	31	58%	8	50%
Man	19	36%	7	44%
Gender queer	2	4%	1	6%
Trans male	1	2%	-	-
**Race/Ethnicity of Participants**				
White	41	77%	14	88%
Asian or Asian American	4	8%	-	-
Black or African American	2	4%	-	-
Hispanic or Latinx	2	4%	1	6%
Prefer Not to Respond	2	4%	-	-
American Indian or Alaska Native	1	2%	1	6%
White; American Indian or Alaska Native	1	2%	-	-
**Age group**				
20–29	1	2%	1	6%
30–39	13	25%	3	19%
40–49	20	38%	5	31%
50–59	13	25%	4	25%
+ 60	6	11%	1	6%
**Credential**				
MD/DO	16	-	5	-
BA/BS/MS	11		3	
LCSW/LPC/NCC	7	-	4	-
Other	5	-	1	-
Nursing	4	-	1	-
MPH	4	-	2	-
PhD	3	-	1	-
PharmD	2	-	-	-
MA	2	-	-	-
NP	2	-	-	-
ND	1	-	-	-
None	1	-	-	-
**Location of Participants**				
Practice in Multnomah^1^ county	36	68%	9	56%
Practice located in rural area as defined by HRSA^2^	7	13%	4	25%
Number of Oregon’s 36 counties represented by ≥ 1 participant	10		7	
**Leadership Level**				
Direct Care	11	21%	2	13%
Organization or Program	22	42%	8	50%
State or Regional Systems	20	38%	6	38%
**Organization Classification**				
Large Health System	13	25%	5	31%
Specialty Addiction and Behavioral Health Treatment	12	23%	2	13%
Primary Care	8	15%	3	19%
Accountable Care Organization	6	11%	2	13%
Advocacy Nonprofit	5	9%	-	-
State Health Authority	5	9%	1	6%
County Health Department	4	8%	3	19%

^1^ Multnomah county includes the city of Portland and is the most populated county in Oregon.

^2^ Health Resources & Services Administration. Information on how HRSA defines rurality: https://www.hrsa.gov/rural-health/about-us/what-is-rural

Overall, participants reported that the SUD Leadership ECHO sessions were beneficial. Three participants were inspired to make practice and policy changes within their systems. However, many leaders who were unable to make changes identified several barriers. Leaders also suggested modifications to improve future SUD leadership ECHO sessions. Below we summarize the results in three themes: SUD Leadership ECHO benefits, practice and policy changes, and recommendations for improving ECHO sessions.

### SUD leadership ECHO benefits

#### Community building across disciplines and organizations allowed for cross-pollination of ideas

Many participants commented that the chosen SUD Leadership ECHO topics were timely and relevant to what was occurring at in their practice or organization. However, when asked directly about the benefits of participation, many did not reference specific topics and needed prompting to remember specific session content. Instead, participants pointed to the multidisciplinary nature of the ECHO and the exchange of information between leaders.

The majority appreciated hearing about pressing SUD treatment and harm reduction topics from multiple perspectives by accessing, engaging, and connecting with a community of thinkers, advocates, and policy makers. This was described as “moving beyond their siloes”, with multiple participants sharing that outside of this group, few opportunities exist for direct interaction with other leaders or colleagues who lead SUD work in Oregon. Participants remarked positively about “having access to a community of thinkers” and that it was valuable “to hear and see what works.”

Participants also appreciated sharing diverse experiential knowledge. This helped them develop new perspectives on complex emerging SUD issues with less concern for gaps in their understanding. For example, one participant (17, State/Regional systems leader) remarked on the importance of having high-level leaders present to understand how things functioned on the administrative and policy side, explaining “[it’s] nice to have people that understand higher up level change and what we need to do and how systems work and not just like, oh, we need to do this, but don’t understand the background of it.” Similarly, another participant (15, Direct Care leader) mentioned the uniqueness of engaging with high level leaders in this setting, saying “I don’t think [the state health authority] comes to any other CME I do.” Other participants (4, Direct Care leader) reflected on the importance of including folks with lived experience with SUD, describing how it “changed some of the conversations that are happening in the community” and that “it [is] beneficial for folks who are in policy, who are in direct care to be hearing about those. So we have more of the spokes present around this shared purpose of people having access to great care.” A fourth participant (14, State/Regional systems leader) commented on how the SUD Leadership ECHO engaged providers beyond physicians, when compared to other societies, saying, “It [State Society of Addiction Medicine], is primarily physicians, and I don’t participate in those activities in general…there’s something really nice about the crosscutting nature of who comes to this ECHO.”

#### Reduction of isolation among leaders

Participants reflected that the SUD leadership ECHO created a communal space where there was shared understanding that SUD treatment and harm reduction work is challenging, with a shared commitment to persevere. Participants explained that SUD leaders can feel uniquely isolated, describing feeling a mission and drive to improve SUD care and policy, while simultaneously feeling fatigued, isolated, or defeated when change doesn’t happen. These sentiments were captured by two participants who said:

For me, being kind of isolated out here and just being able to sit in a room full of other people grappling with some of the same issues, efforts to implement in the hospital and the million harm reduction things that we would, could, should be doing and are struggling to try to implement in a conservative community, just having the space to come and be with other folks has been of massive benefit to me. - Participant 8 (State/Regional systems leader).

Being in community with people who already understand that it’s hard… In a room of normy’s explaining the work we do and why it’s important, is a lot of work. And so being able to be with this group of people who I don’t have to explain why what I do is important. - Participant 16 (Organization/Program leader).

#### Focus on local implementation

Participants observed that the focus on local implementation was unique and informed them of policies and practices taking effect in real time across the state. This was cited as different from other learning communities, conferences, or SUD focused groups that participants were a part of, which focused on more didactic approaches, and were slower to provide relevant information. One participant (8, State/Regional Systems leader) clarified the value of shared systems context: “I always enjoy the fact that it’s folks from around our state specifically who just have to deal with the same policies and governance structures and understand how things operate in Oregon.” Another participant (7, Organization/Program leader) explained, “I hear and I learn a lot about what’s going on in other parts of the country, but then for the, ‘well, how does that translate to Oregon, or are we doing….’ And these forums seem to be a great way to learn about what’s happening in Oregon and where I might be able to connect with somebody here.”

### Practice and policy changes as a result of participation in SUD leadership ECHO

#### Leaders who successfully made changes heard best practices and how other organizations approached problems

Three participants mentioned making practice or policy changes; two were Organization & Program leaders and one was State & Regional leader. Changes included the creation of a two-day training to support changes in Suboxone prescribing policy, updates to staff engagement and retention operational policies, and partnering with the local jail to initiate Suboxone prescribing. All three described drawing inspiration from the exchange of ideas and being influenced by the approaches of others. Two of these participants felt that adopting a model tried successfully by others within the state helped convince stakeholders in their home organizations to embrace changes. Participants placed a high importance on gaining insights for real-life implementation in complex contexts, echoing findings from the previous section. One participant (3, Organization/Program leader) shared that their position specifically empowered them to make changes incited by the ECHO; program and policy implementation was an explicit part of their role. This participant also had a clinician partner enrolled in a separate addiction medicine ECHO. Their collaboration was instrumental in driving change within their organization:

And I think my doctor partner, she had the same experience [in a different SUD ECHO] where she’s like, ‘Why are we doing this, this way? I’m hearing from these other leaders, they’re doing it this other way.’ And so between the two of us, we were able to really influence our teams. (Participant 3, Organization/Program leader).

#### Barriers to initiating practice and policy changes included lack of formal leadership authority, time constraints, and systemic issues

In contrast, five others reported no practice or policy changes. One participant self-identified as merely an informal leader in their organization, and felt they lacked power to initiate changes. They “wonder(ed) if that [informal vs. formal leadership role] correlates to who feels they can make change.” (Participant 15, Direct Care leader) Participants further explained that policy changes take time, often longer than a year or two, so changes might not have occurred in the time span of this evaluation. A few participants recognized that high level systems obstructed the possibility of organizational-level changes, such as pharmacy-level buprenorphine shortages or state or federal-level policy restrictions. For example, one participant (8, State/Regional Systems leader) described that although practices they heard about in the ECHO sessions were in alignment with conversations they were having in their organization, changes “are not feasible just yet for typically a variety of policy reasons.”

Even for participants who did not report making changes, ECHO may have catalyzed future developments. One participant (11, Organization/Program leader) encapsulated this saying, “Even though I can’t think of a direct way that it led to any specific changes, it does stimulate conversation and ideas to start going.”

### Recommendations for improving SUD leadership ECHO sessions

#### Tension existed between wanting to explore local implementation and national SUD context

While the focus on Oregon-centric policy and implementation was identified as a strength, some participants acknowledged that a balance of perspectives from the national landscape would help explore new ideas. As one participant (7, Organization/Program leader) described, “As much as I really enjoy being able to learn about what’s happening in Oregon and find a lot of value there. There are things that are happening in other parts of the country that aren’t happening here… To spark the conversation of, ‘What does that look like in Oregon?’”.

#### Focused presentations on one issue from multiple perspectives were desired as opposed to the traditional ECHO structure of two topics (case and didactic) in one session

The criticism raised most often was that there was too much content in each 1-hour session. Participants felt that “we were trying to squeeze a lot into a small amount of time…the topics chosen were not quick, easy.” Discussions were robust, but “we had to pivot either to the next topic or to the end of the time when there was still some really great idea generation and discussion happening that I would’ve loved to like been able to continue with” (Participant 8, State/Regional systems leader). To remedy this, one participant (2, State/Regional systems leader) suggested, “to have multiple different type of experts or people with different types of experiences talk about … the same topic.”

#### Greater focus on solutions, advocacy, and next steps

Discomfort at the close of ECHO sessions was also tied to a desire to focus on solutions, particularly as participants felt this group had the expertise to solve the problems discussed. There was agreement about dysfunctional systems but the discussion yielded “no tangible next step” (Participant 3, Organization/Program leader). One participant (14, State/Regional Systems leader) commented that the lack of “real accountability” was “a limitation of the Leadership ECHO” even though it gave “visibility and transparency where the problems are.” Time was a barrier to developing solutions; one participant (16, Organization/Program level leader) expressed, “this leadership ECHO is really more solutions based, than it actually got to be. We talked about a lot of problems, and then ran out of time before we could discuss solutions.”

The participants were interested in further contributing to advocacy. A few suggested the ECHO faculty team could: 1) facilitate petitions, 2) create intentional sub-committees to develop changes postulated during the ECHO, and 3) add accountability by tracking follow-through between sessions. On the theme of sub-committees, participants were interested but unsure how to organize. As one participant (5, State/Regional Systems leader) pointed out “Some folks are going to be more invested in certain ideas or areas of change and have more experience or insights into it” but may not have “an ability to commit to a timing around it.” However, participant 3 (Organization/Program leader) described leveraging the collective power of the leaders present, “It just doesn’t make sense that we’re raising our voices in silos. It would be much more powerful if we all put our voices together and had some sort of shared mission agenda something in writing that said, ’This is what our community SUD Leadership wants.’”.

## Discussion

Participants emphasized that the greatest benefit was connecting with other leaders with diverse perspectives who were dealing with similar issues in their organizations, and that these connections reduced isolation. Addiction treatment providers may be at heightened risk for burnout as they support a patient population with highly complex social and behavioral needs with significant trauma [18–20]. Prior research has described social support as a key antidote to improving resilience and reducing burnout for healthcare providers [21, 22]. New evidence suggests that for addiction medicine providers, specifically, enhancing their ability to advocate and engaging with others across institutions to enact higher-level change may improve satisfaction and increase resiliency [20]. Our data shows that the SUD leadership ECHO empowered and connected leaders, and suggests that the community built by these sessions may represent one avenue to reduce burnout in these highly susceptible leaders.

Notably, unlike prior evaluations of ECHOs, the presented content was not named as a reason for attending [14, 23, 24]. In fact, the majority of participants could not recall specific session topics without prompting. This was particularly surprising for two reasons. First, in the typical ECHO model, the didactic topics form the backbone of the program and curriculum topics are advertised to promote attendance. Second, for the SUD Leadership ECHO, extra effort was invested to curate attractive and timely content. When the ECHO was repeated, the faculty team sought out brand new presenters and topics to keep the sessions relevant. These findings indicate that ECHO participants in leadership roles may have different needs than the typical medical provider population that ECHO traditionally serves, and special attention may need to be paid to the interactive, networking, and mutual-support aspects to provide value.

Leaders also highlighted several limitations of the ECHO. Sessions featured two topics in a single hour at the expense of depth. Cohorts 1 and 2 of the SUD Leadership ECHO held the traditional ECHO adult learning model of didactic and case studies within each session. The ECHO’s faculty team hoped to promote attendance by using a familiar model, but around half of program participants were brand new to ECHO programming. A second goal was to provide high-yield sessions by hosting condensed but meaningful discussions on two topics per session. The faculty team tried to achieve this vision by preparing extensively, coaching presenters to distill their information, and actively facilitating. However, the didactic and case model left many unsatisfied since the topics covered were complex and it was difficult to explore them adequately in 30 min. In light of these findings, the faculty team adapted to single topic sessions with panel presentations for Cohort 3. This marks a significant diversion from the typical ECHO model but promises to better meet the needs of the unique audience and uniquely complex topics; future work will assess the tradeoffs of this shift in content and structure.

Facilitating policy change was a primary goal of the SUD Leadership ECHO, however, participants often could not make changes directly. Networking and information sharing were sometimes adequate for participants to make changes within their own organizations. Participants reported this worked best when the problem was primarily unsolved because it was new to the organization. Borrowing good ideas from SUD leadership ECHO colleagues was less effective if problems were systemic in scope, needed higher level action to achieve change, or necessitated new legislation. Additionally, there was limited follow-up assistance provided by ECHO to carry change ideas forward. In Cohort 3, responding to this feedback, the ECHO hosted the advocacy officer for the American Society of Addiction Medicine and facilitated participants to comment during the rulemaking process for federal changes to methadone regulation [25].

While the number of participants who initiated organizational changes was small, two key factors were identified that helped support changes. First, those able to initiate changes typically had a leadership role with access to organizational operations. This is well-supported, as policy and implementation change typically require adaptations to organizational contexts and financial resources which direct care providers may not have authority over [26, 27]. Second, one participant illustrated that having like-minded colleagues within their organization allowed them to build a stronger case for change. Generally, only one person per organization attended the SUD Leadership ECHO; the faculty intended through careful recruitment to maximize diversity in the program rather than recruit multiple leaders per organization. However, this approach may have undermined the goal of organizational-level change.

Participants also requested more avenues to generate solutions during and after the ECHO sessions. Suggestions included facilitated petition letters, sub-committee formation, and follow up in the full ECHO forum to ensure accountability. These suggestions highlight that the greatest barrier to progress may not be lack of time, even though conversations felt unfinished. Instead, change is a longitudinal process and effective advocacy is challenging, even for this group of experienced leaders. Promoting longitudinal changes is outside the scope of the traditional ECHO, which suggests the need to divert from the traditional model when designing sessions for this unique population. Future iterations of the SUD leadership ECHO could include adding an experienced advocate to the ECHO faculty team, or partnering with advocacy organizations to facilitate advocacy activities.

Our study has a few limitations. First, it might be affected by selection bias. Participants who were more significantly influenced by the ECHO and engaged in Oregon’s addiction medicine ECHO program may have been more likely to enroll in the focus groups. This could have resulted in a more positively biased evaluation of the ECHO. Second, the evaluation of changes was constrained by our timeframe. We conducted our post-program evaluation two months after the ECHO sessions were completed. As noted by several participants, organizational change can take months to years, and we may not have captured all practice and policy changes that will be made in response to participation in this ECHO. Future work could explore whether this novel ECHO model was successful in driving leaders to initiate changes within their organization over a lengthier time frame. Despite these limitations, our study provides valuable insight into this novel adaptation of the ECHO model, its drawbacks, and how it uniquely met needs for SUD care leaders.

## Conclusion

Overall, the SUD Leadership ECHO was well received by focus group participants, with mixed reports on whether initiating organizational or policy change was achieved. This study confirms that replication of the SUD Leadership ECHO model, with fine-tuning, is promising. The feedback provided shows that there is untapped potential in branching away from the traditional ECHO model to meet the unique needs of SUD care leaders. Further research is needed to understand how SUD care leaders are served by ECHO versus their national professional societies and local chapters and assess if ECHO is the best forum to fill these gaps and meet SUD care leader’s needs. Furthermore, future studies should identify the avenues and program structures that are most beneficial to support organizational and local-regional SUD leaders in promoting systems change to improve substance use care.

## Appendix A: Focus group guide

### Introductory script

Thank you for joining our Focus Group today. We are interested in learning the ways in which participants benefited from their involvement in the SUD Leadership ECHO; whether and what SUD practices and/or policies changed as a result of their participation in the SUD Leadership ECHO; participants’ recommendations for improving the SUD Leadership ECHO; and whether and what opportunities participants see for group advocacy around improving SUD prevention, harm reduction, recovery, and supporting people with SUD in Oregon.

This work is funded by the SUD leadership ECHO program, however, we will not share specific details about you (e.g., your name, clinic) or directly link you to your responses when reporting findings back. We are hoping to publish a paper from these findings so that others replicate some of the successful practices that were developed here.

Today, I will be acting as the facilitator of this focus group—I will be presenting the topic areas and probing for any follow-up details. I am joined by [ECHO Program Lead], [Qualitative Analyst], [ECHO project coordinator], who are here to take notes, help me keep track of time, and monitor the chat.

A focus group differs from an interview in that we are interested in your discussions around the topic. We encourage you to interact and discuss with each other. There are no right or wrong answers, so please share your experiences and thoughts as we continue.

We would like to record this focus group so we can accurately capture your experiences in your own words. This recording will be transcribed and all proper names and places will be removed to protect your identity and privacy. Do I have permission to audiotape this focus group?

Great, thank you. I may ask at times to clarify who’s speaking to make sure I’m tracking your responses. If you agree with what others are saying, please feel free to emphasize this in your replies!

Before we dive in to our questions, I want to share ground rules for today’s conversation: Before we get started we would like to remind you that everything said here should remain confidential. Stories shared here should not be shared outside of the group. Second, we would like to hear from all participants. If you are someone who finds yourself speaking up a lot, please remember to step back at times to let others speak. On the contrary, if you are someone who finds yourself listening and observing, please step up and share your experiences, it’s extremely valuable for us to hear all opinions, especially if they are different from the majority. Any questions regarding these?

## Introduction

1. Please share your name, organization, role, in the chat.

**Feedback on structure and content of ECHO**

2. How were you connected to the SUD leadership ECHO?

- Probe: What motivated you to first attend the SUD leadership ECHO sessions?
- Probe: Why did you continue attending the SUD Leadership ECHO sessions?

3. What were the benefits of your involvement in the SUD Leadership ECHO program?
4. Which sessions were most impactful? Why?

- Probe: Didactic/ SBAR, structural pieces, what the amount of content just right, not enough, or too much.

5. What new professional connections did you make? How has this impacted your current practice?
6. What new resources did you access? How has this impacted your current practice?
7. What sets the SUD leadership ECHO sessions apart from other forums (conferences, CME training, etc.)?
8. What made it easy to participate in the SUD Leadership ECHO program? What went well?
9. What opportunities are there for the SUD Leadership ECHO to be improved?

- Probe: What changes could be made to how the sessions are facilitated?
- Probe: What changes could be made to the structure of the ECHO?
- Probe: What additional strategies could be used to build relationships and make the sessions interactive?
- Probe: Was anyone missing from the conversation? If so, who should be invited to these conversations? (specific as possible: ex. name, organization, rationale for inclusion)

10. Are you continuing to participate in the SUD Leadership ECHO? Why or why not?
11. Why didn’t you attend more sessions?

**Practice and/or Policy Changes**

12. Did anything about your current practices change as a result of participating in the SUD Leadership ECHO?

- Probe: If so, please describe the specific actions you took to make such changes.

13. Has your organization changed – or started to think about changing – any policies or practices as a result of your participating in the SUD Leadership ECHO?

- Probe: If so, please describe specific actions your organization as took or plans to take to make such changes.

**Opportunities for Systems Change and Advocacy**

14. Do you see any opportunities for how SUD Leadership ECHO participants may advocate for systems change around SUD prevention, harm reduction, treatment or better supporting populations with SUD?

- Probe: What areas do you see opportunities and what could that advocacy look like?
- Probe: If not, please describe barriers to group collaboration and advocacy.
- Probe: How could the ECHO support in making these changes? (e.g., what is already happening, what could be happening?)
- Probe: How else can ECHO support systems changes and advocacy (e.g., letters to certain parties)?

## Conclusions

15. Is there anything else you’d like to share?

Thank you for participating in the focus group today and your candidness in discussing these topics related to SUD. Your responses will be used to inform future ECHOs around SUD. Don’t hesitate to reach out to us if things come to mind after this focus group. We can incorporate those thoughts into our analysis, too.

Thank you so much, and enjoy the rest of your [day].

## Data Availability

The datasets generated and/or analysed during the current study are not publicly available due privacy protection of participants.

## Abbreviations

CME: Continuing medical education
ECHO: Extension for community healthcare outcomes
OEN: Oregon echo network
OHSU: Oregon health & science University
SUD: Substance use disorder
